# Illustrated Manual of Operative Surgery and Surgical Anatomy

**Published:** 1852-08

**Authors:** 


### BIBLIOGRAPHICAL NOTICES.

*Illustrated Manual of Operative Surgery and Surgical Anatomy.* By MM. Cl. Bernard and Ch. Huette. *Edited with notes and additions, and adapted to the use of the American Medical Student, by* W. H. Van Buren, M. D., Surgeon to Bellevue Hospital, &c., and C. E. Isaacs, M. D., Demonstrator of Anatomy in the College of Physicians and Surgeons, New York. *Illustrated with steel engravings from drawings after nature, by* M. J. Léveillé. *Designed to serve as a companion to the ordinary text-books of Surgery.* New York: H. Bailliere, 290 Broadway. 1852. Part II. (pp. 188 and 27 plates.)

The second part of the American translation of Bernard and Huette's manual maintains in all respects the character established by the first as an excellent reproduction of the French original, and as a beautiful and instructive atlas of Operative

Surgery. It cannot fail to prove a useful adjunct to the different works on Operative Surgery adopted in our schools, and thus handsomely to serve the modest purpose for which it is especially offered to the profession, whilst it may be resorted to with advantage by teachers and practitioners, even in the absence of the text-books.

The present volume begins with eight preliminary plates representing respectively and successively the instruments employed in trepanning, in operating on the eye and the ear, on the larynx and trachea, in the treatment of nasal polypi, in operating on the tonsils and soft palate and on the pharynx and larynx. After these comes Plate 27th, on amputation at the hip joint, in regular continuation of the articular amputations. This is followed by four plates devoted to amputations in the continuity of limbs, beginning with amputations of the foot and hand and ending with amputation of the thigh. Then we have three plates of exsections of the superior and inferior extremities and of the upper and lower jaw. These are succeeded by one on trepanning of the bones of the cranium, (including, as usual, the surgical anatomy of the parts involved in the operation); two on operations on the eyelids; two on the surgical anatomy of the lacrymal apparatus and of the nasal fossæ, the mouth and the pharynx, with the operations performed upon their different ducts and passages; one upon the surgical anatomy and operations relating to the muscles of the eye; one delineating the operation for cataract by depression; another representing the different stages of the operation for extraction, and the various methods of operating for artificial pupil; another representing operations upon the external and internal ear and the surgical anatomy of the parts concerned; two plates devoted to hare-lip, cheiloplasty and other autoplastic operations on the orifice of the mouth; and lastly, by one plate (46th) on rhinoplasty.

It will be seen from the foregoing cursory list that the editors have furnished us in their last instalment (small though it be and beautifully less in comparison with their first) with many of the most important and interesting of the entire series of illustrations. The accompanying text is well arranged, clear, concise and sufficiently full for the purpose intended; and appears to be elegantly and accurately rendered into our vernacular. On every account therefore we shall hail with interest the speedy completion of the work. It would give us sincere pleasure to view (and *re*-view) our friends' production as a whole—especially in colors. As to these latter we are yet in darkness. Nos. 1 and 2 have come to us in mourning—shorn of half their glory. We trust that the forthcoming portions will throw a better light upon the subject so far as we may be concerned. In this matter of reviewing we confess to some weakness for the "feathers" along with the "fuss"; and when we have to introduce a book to our readers, we expect to see it in the best dress that belongs to it.

